# Temporal Relationship between Impairment of Cerebellar Motor Learning and Deterioration of Ataxia in Patients with Cerebellar Degeneration

**DOI:** 10.1007/s12311-023-01545-1

**Published:** 2023-04-28

**Authors:** Takeru Honda, Ken Matsumura, Yuji Hashimoto, Takanori Yokota, Hidehiro Mizusawa, Soichi Nagao, Kinya Ishikawa

**Affiliations:** 1 Department of Neurology and Neurological Science, Graduate School, Tokyo Medical and Dental University, 1-5-45, Yushima, Bunkyo-Ku, Tokyo 113-8510 Japan; 2 Basic Technology Research Center, Tokyo Metropolitan Institute of Medical Science, 2-1-6 Kamikitazawa, Setagaya-Ku, Tokyo 156-8506 Japan; 3 Laboratory for Higher Brain Function, Nozomi Hospital, Ina, Kitaadachi-Gun, Saitama, 362-0806 Japan; 4 The Center for Personalized Medicine for Healthy Aging, Tokyo Medical and Dental University, 1-5-45, Yushima, Bunkyo-Ku, Tokyo 113-8510 Japan; 5 Department of Neurology, Tokyo Metropolitan Komagome Hospital, 3-18-22 Honkomagome, Bunkyo-Ku, Tokyo, 113-8677 Japan; 6 Department of Internal Medicine, Tokyo Metropolitan Matsuzawa Hospital, 2-1-1 Kamikitazawa, Setagaya-Ku, Tokyo 156-0057 Japan; 7 National Center Hospital, National Center of Neurology and Psychiatry, 4-1-1 Ogawahigashicho, Kodaira, Tokyo 187-8551 Japan

**Keywords:** Motor learning, Prism adaptation, Ataxia, Cerebellar degeneration, Disease duration, SARA

## Abstract

Ataxia and impaired motor learning are both fundamental features in diseases affecting the cerebellum. However, it remains unclarified whether motor learning is impaired only when ataxia clearly manifests, nor it is known whether the progression of ataxia, the speed of which often varies among patients with the same disease, can be monitored by examining motor learning. We evaluated motor learning and ataxia at intervals of several months in 40 patients with degenerative conditions [i.e., multiple system atrophy (MSA), Machado–Joseph disease (MJD)/spinocerebellar ataxia type 3 (SCA3), SCA6, and SCA31]. Motor learning was quantified as the adaptability index (*AI*) in the prism adaptation task and ataxia was scored using the Scale for the Assessment and Rating of Ataxia (SARA). We found that *AI* decreased most markedly in both MSA-C and MSA-P, moderately in MJD, and mildly in SCA6 and SCA31. Overall, the *AI* decrease occurred more rapidly than the SARA score increase. Interestingly, *AI*s remained normal in purely parkinsonian MSA-P patients (*n* = 4), but they dropped into the ataxia range when these patients started to show ataxia. The decrease in *AI* during follow-up (*dAI/dt*) was significant in patients with SARA scores < 10.5 compared with patients with SARA scores ≥ 10.5, indicating that *AI* is particularly useful for diagnosing the earlier phase of cerebellar degeneration. We conclude that *AI* is a useful marker for progressions of cerebellar diseases, and that evaluating the motor learning of patients can be particularly valuable for detecting cerebellar impairment, which is often masked by parkinsonisms and other signs.

**Supplementary Information:**

The online version contains supplementary material available at 10.1007/s12311-023-01545-1.

## Introduction

Cerebellar damage causes ataxia [1], which includes disturbances in balance, speech, and coordination of limb movements. Neurologically, ataxia is often quantified using the International Cooperative Ataxia Rating Scale (ICARS) [2] or the Scale for the Assessment and Rating of Ataxia (SARA) [3]. Although both are subjective rating methods, several neurophysiological studies using a paradigm of adaptations of forelimb movements have consistently suggested that patients with cerebellar degenerations show a deficit in motor learning [4–7]. However, how the impaired motor learning is correlated with ataxia and how cerebellar motor learning deteriorates with the duration of the cerebellar disease have not yet been clarified.

Prism adaptation [4–6, 8] is often used to quantify human motor learning capability. In prism adaptation, the subjects wearing the prism, which considerably shifts the visual field, are instructed to touch with their finger the target presented in front of them. Healthy subjects can quickly learn how to precisely touch with their finger the target, even when their gaze is artificially shifted rightward or leftward by the prism lens that the subjects wear. In our previous study, we quantified motor learning capability by calculating the adaptability index (*AI*). Whereas the *AI*s of healthy subjects before 70 years of age were typically ≥ 0.68, the *AI*s of the patients with cerebellar degeneration were lower than 0.68 [5]. Furthermore, by comparing *AIs* with SARA scores, we found a negative correlation between them. However, it has not been clarified whether *AI*s be used to detect a cerebellar disease from its early phase. In addition, it remains unclarified whether the *AI* is sensitive for evaluating disease severity. To address these questions, we repeatedly measured the *AI*s and SARA scores of 40 patients with cerebellar degenerations at intervals of more than three months. We compared changes in *AI*s and SARA scores to determine whether the impairment of motor learning starts earlier than the progression of ataxia.

## Materials and Methods

### Participants

We recruited 40 patients with cerebellar degeneration (mean age, 55.4 years; range, 31–68 years). Among them, 16 had multiple system atrophy with predominant cerebellar ataxia (MSA-C) and five had multiple system atrophy with predominant parkinsonism (MSA-P). MSA was diagnosed in accordance with the 2^nd^ consensus statement of MSA [9]. Four MSA-P patients (CD17, CD18, CD19 and CD21) initially presented with pure parkinsonism and autonomic dysfunctions defined as “pure parkinsonian” MSA-P. For spinocerebellar ataxias (SCAs), nine patients with spinocerebellar ataxia type 3/Machado-Joseph disease (MJD), five patients with SCA6, and another five patients with SCA31 were studied (Table 1). These SCA diagnoses were confirmed by genetic testing. Disease duration was expressed in months, defined as the duration from the disease onset, that is, when the patients first noticed the signs of their disease, until the time of examination. The first test of 28 of the 40 patients who participated in this study was carried out in our previous study [5].

**Table 1:** Characteristics of patients with cerebellar disease

Patient	Age at 1st test/ Gender	Diagnosis	Age at onset	Disease Duration (Month)	AI	SARA
CD1	63/F	MSA-C	61	25	0.064	10.5
				36	0.288	14.5
CD2	56/M	MSA-C	54	27	0.486	7
				35	0.216	7.5
				39	0.112	11.5
CD3	62/M	MSA-C	56	76	0.096	11.5
				82	0	13
CD4	56/M	MSA-C	52	45	0.378	12.5
				50	0.196	13
				54	0.24	12
				63	0.256	15
CD5	60/M	MSA-C	58	33	0.14	8
				42	0.18	12
CD6	61/M	MSA-C	56	57	0.108	23.5
				63	0	23.5
CD7	56/M	MSA-C	53	40	0.168	15
				46	0.144	18
CD8	58/M	MSA-C	57	19	0.144	12
				27	0.16	19
CD9	57/M	MSA-C	55	51	0.384	6.5
				61	0.384	5.5
				77	0.288	8
CD10	64/M	MSA-C	61	36	0.324	8
				44	0.098	9
CD11	58/M	MSA-C	56	28	0.504	13.5
				36	0.324	13.5
CD12	52/F	MSA-C	50	29	0.486	9
				33	0.56	12.5
CD13	54/M	MSA-C	49	44	0.16	10
				53	0.252	15.5
CD14	48/M	MSA-C	47	9	0	13.5
				15	0.1	16.5
				19	0	17.5
CD15	66/F	MSA-C	64	25	0.112	11
				33	0.168	17
CD16	68/M	MSA-C	59	99	0.024	12.5
				105	0.064	11
CD17	65/M	MSA-P	63	20	0.54	7.5
				33	0.36	10
				37	0.288	13
				44	0.256	15
CD18	63/M	MSA-P	62	16	0.64	10
				22	0.216	13.5
				26	0.18	16.5
CD19	56/M	MSA-P	52	42	0.8	1.5
				46	0.648	1.5
				52	0.36	5
				61	0	13.5
CD20	64/F	MSA-P	61	38	0	10
				45	0	14
CD21	65/M	MSA-P	62	38	0.6	6.5
				49	0.2	8
CD22	48/M	MJD	41	90	0.126	14.5
			–	110	0.096	17.5
CD23	48/M	MJD	36	159	0.336	24
				168	0.12	25
				184	0.096	25
CD24	36/F	MJD	30	72	0.324	16
				81	0	13.5
CD25	31/F	MJD	27	51	0.36	7.5
				60	0.064	9
CD26	46/F	MJD	43	36	0.48	9
				44	0.072	8
CD27	45/M	MJD	37	85	0.42	6
				92	0.48	6.5
				101	0.24	5
				108	0	10
CD28	41/F	MJD	20	269	0.168	14.5
				275	0.03	17
CD29	46/M	MJD	32	163	0.64	7
				170	0.288	10
CD30	33/M	MJD	27	75	0.48	11.5
				81	0.18	11.5
CD31	63/M	SCA6	51	153	0.012	26
				167	0	26
				174	0	27.5
CD32	39/M	SCA6	29	114	0.36	10
				122	0.486	8
CD33	62/F	SCA6	49	147	0.32	14
				153	0.192	16
CD34	62/M	SCA6	50	142	0	14
				151	0	17
CD35	47/M	SCA6	42	57	0.36	13
				72	0.28	21
CD36	66/F	SCA31	51	182	0	13.5
				187	0.06	13.5
				193	0	16.5
CD37	63/F	SCA31	56	96	0.54	10.5
				103	0.48	15
CD38	68/M	SCA31	59	104	0.144	11
				117	0.36	12
CD39	65/M	SCA31	58	94	0.64	10
				99	0.32	10.5
				112	0.09	9.5
CD40	56/F	SCA31	51	60	0.4	10.5
				66	0.18	9.5

*CD* patients with cerebellar disease, *MSA-C* multiple system atrophy with predominant cerebellar ataxia, *MSA-P* multiple system atrophy with predominant parkinsonism, *MJD* Machado–Joseph disease, *SCA6 and SCA31* spinocerebellar ataxia types 6 and 31, respectively, *M* male, *F* female, *SARA* Scale for the Assessment and Rating of Ataxia, *AI* adaptability index

After obtaining written informed consent, they were repeatedly evaluated to monitor their disease state and prism adaptation at intervals of more than three months. Twenty-eight patients were evaluated twice, whereas the remaining 12 patients were evaluated more than twice, yielding 56 intervals totally. Their visual acuity was normal or corrected with spectacles. The experimental procedure was approved by the Ethics Committee of Tokyo Medical and Dental University.

### Experimental Apparatus and Task

Details of the experimental apparatus and procedure were described previously by Hashimoto et al. [5]. Two Windows 7 personal computers, one for the task control and the other for data sampling and analysis, with a 23-inch touchscreen monitor were used.

**Fig. 1:**
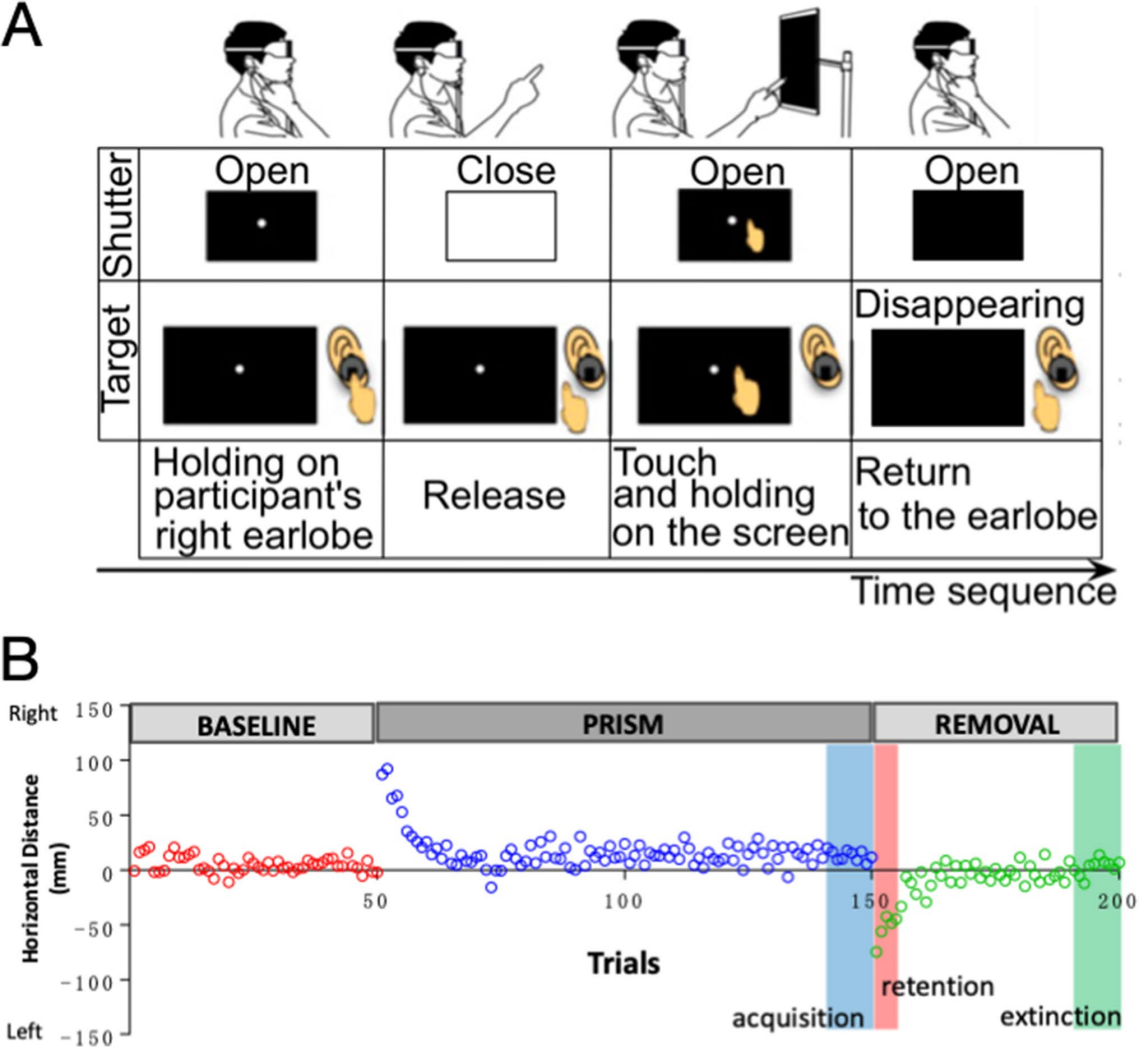
Scheme for the prism adaptation of hand-reaching movement. (**A**) Prism adaptation task. The time sequence of a single trial is shown from left to right. Every trial starts from the time a participant’s index finger touched the sensor attached on the right earlobe. As soon as the participant releases the index finger from the sensor, vision is blocked by the electrical shutter. Immediately after touching the touchscreen, the goggles become transparent, and the participant recognizes whether the index finger deviated or hit the target for 100 ms (ms). Subsequently, the target is extinguished and the index finger returns to the original position to prepare for the next trial. (**B**) An example of a healthy subject’s finger-touch errors represented by the horizontal distance (mm) from the target to the touch point with trials. He was 36 years old. Positive and negative values indicate the degree of rightward and leftward shifts, respectively. The abscissa shows the number of trials. Acquisition, retention, and extinction are shown in blue, red, and green square areas, respectively. The *AI* of this subject is 1.0

The participants quietly sat on the chair in front of the touchscreen monitor in a dark room, wearing the custom-made goggles with their chin on a chin-rest. A sensor was attached to their right earlobe. When the participants touched the earlobe sensor with their right index finger, a target (8 mm-diameter white circle) appeared at a position randomly selected on the touchscreen monitor. Then, the participants were instructed to touch with their right index finger the target. After the participants released their index finger from the earlobe sensor, the electrical shutter mounted on the goggles was closed to prevent the participants from tracking the target with their right finger intentionally. When their finger touched the touchscreen, the shutter was reopened, which enabled the participants to confirm the positions of their finger and the target. Then, the target disappeared again to initiate the next trial (Fig. 1A).

There are three sessions: the first 50 trials with normal vision wearing a transparent plastic plate (BASELINE), the second 100 trials wearing the prism that shifts the visual field 25 degrees rightward (PRISM), and the third 50 trials wearing the transparent plastic plate without the prism (REMOVAL) (Fig. 1B).

### Data Analysis

We calculated the *AI* as described in our previous study [5]. We defined that a participant correctly touched the target when the distance between positions of the target and the right index finger was less than 25 mm on the panel. We calculated the probability of correct touches in the last 10 trials of PRISM (acquisition of adaptation in a blue square area shown in Fig. 1B), that of incorrect touches in the initial five trials of REMOVAL (retention of adaptation in a red square area shown in Fig. 1B), and that of correct touches in the last 10 trials of REMOVAL (extinction of adaptation in a green square area shown in Fig. 1B). Then, we defined *AI* = (*acquisition of adaptation*) × (*retention of adaptation*) × (*extinction of adaptation*).

When the participant’s capability of motor learning is normally maintained, the value of *AI* is 1 (the maximum value). On the other hand, when the participant’s capability of motor learning is severely impaired, *AI* is around 0 (the minimum value). As *AI* decreases with the severity of ataxia, the SARA score increases. To overcome this inverse relationship and to simply compare *AI* with SARA score, we flipped the score of SARA, as SARA index (*SI*) calculated as

1 $$SI = 1-SARA / 27.5,$$

where *SARA* is the score of SARA. When *SARA* is zero, indicating a non-ataxic state, *SI* is 1. On the other hand, when *SARA* is 27.5, representing the severest ataxia condition in the present cohort, *SI* is 0. In this study, we defined *SI* using the maximum SARA score in the present cohort (*SARA* = 27.5) in order to set the ranges of *SI* and *AI* in the same scale 0 to 1. We additionally calculated *SI* assuming that *SARA* can range from 0 to 40.

### Statistical Analyses

The progression rate of degenerative disease is not clearly understood, and a large number of samples are needed to obtain accurate progression rate. Given that numbers of patients and time-points are both limied, we instead aimed to compare the progression rates of different disease conditions by applying an exponential curve for all disease groups. In this regard, we here hypothesized that *AI* and *SI* both monotonically and exponentially decrease from one (maximum) to zero (minimum) with time. These indexes were fitted by the least squares method as follows:

2 $$AI = \mathrm{exp} (-t/a),$$

3 $$SI = \mathrm{exp} (-t/b), \mathrm{and}$$

4 $$AI =\mathrm{ exp }[(SI-1)/c],$$

where *t*, *a*, *b*, and *c* are the disease duration (in months) since the onset defined as the age when the patients first noticed the signs of cerebellar ataxia, time constant of *AI*, time constant of *SI*, and (1 − *SI*) when *AI* decreases from *AI* = 1 to *AI* = exp (− 1) = 0.368, respectively.

In order to assess whether *AI* changes more significantly in earlier ataxia phase than in later phase, the paired-sample *t*-test was used to assess the differences in the change rate of *AI* represented as *dAI/dt* between patients with SARA scores higher than 10.5 and those with SARA scores lower than 10.5. The cut-off SARA score of 10.5 was chosen because the same numbers of halves [total 56 intervals, n = 28 intervals with SARA scores < 10.5 *versus* n = 28 intervals with SARA scores ≥ 10.5 (Table 1)] could be analyzed, whereas sample numbers would deviate if other SARA scores were focused on. *p* < 0.05 obtained by the *t*-test was regarded as statistically significant.

Matlab software (Matlab2013a, MathWorks, MA, USA) was used for these statistical analyses. Data are presented as mean ± standard deviation (SD).

## Results

### AIs in Patients with Cerebellar Diseases

The healthy subjects were able to precisely touch the targets appearing on the screen during the BASELINE session (Fig. 1B). In the PRISM session, they wore a prism that produced a rightward, 25 degrees gaze-shift of the target. Thus, the initial touch point always deviated far right from the actual target. However, the deviation gradually decreased with repetition of the trial, which indicated that the adaptation had occurred. When the prism was removed (the REMOVAL session), the initial touch point shifted far left from the target, as the memory of adaptation was retained. However, soon the memory was extinguished, and the touch points hit the target correctly. We previously reported that the *AI*s of healthy subjects younger than 70 years were between 0.68 and 1.0 [5].

On the other hand, the *AI*s of patients with cerebellar diseases were significantly lower than those of healthy subjects [5]. In agreement with our previous observations, the *AI*s of our 40 patients were below 0.68 (Table 1). The exception for this was noted in one MSA-P patient (CD19) in whom the *AI* in the initial prism adaptation test was 0.8. This patient showed no cerebellar sign (SARA score = 1.5), suggesting a purely parkinsonian state. A follow-up test undertaken four months later also showed an *AI* of 0.648 and a SARA score of 1.5, confirming the patients purely parkinsonian state. However, 10 months after the initial examination, this patient started to show mild cerebellar signs, supported by a SARA score of 5. The *AI* at this point dropped to 0.36, which is within a range indicating the ataxic state. Nineteen months after the initial examination, the patient exhibited obvious cerebellar signs, at which time, the patient’s SARA score was 13.5 and the *AI* was 0. Three other MSA-P patients with very subtle cerebellar signs (CD17, CD18, and CD21) also showed relatively higher *AI*s (0.54, 0.64, and 0.6, respectively) at initial examinations (Table 1).

Among the 56 intervals that we tested, a majority of intervals (42 intervals; 75% of all examinations) showed *AI* decrease, which was consisted with the progression of their diseases. We observed 14 intervals where the follow-up *AI* increased compared to the former *AI*. Notably, the increase was very small; it ranged from 0.016 to 0.224 with a mean value of 0.083 (SD = 0.065).

### Example of Changes in AI and SI with Cerebellar Disease Duration in a Patient

**Fig. 2:**
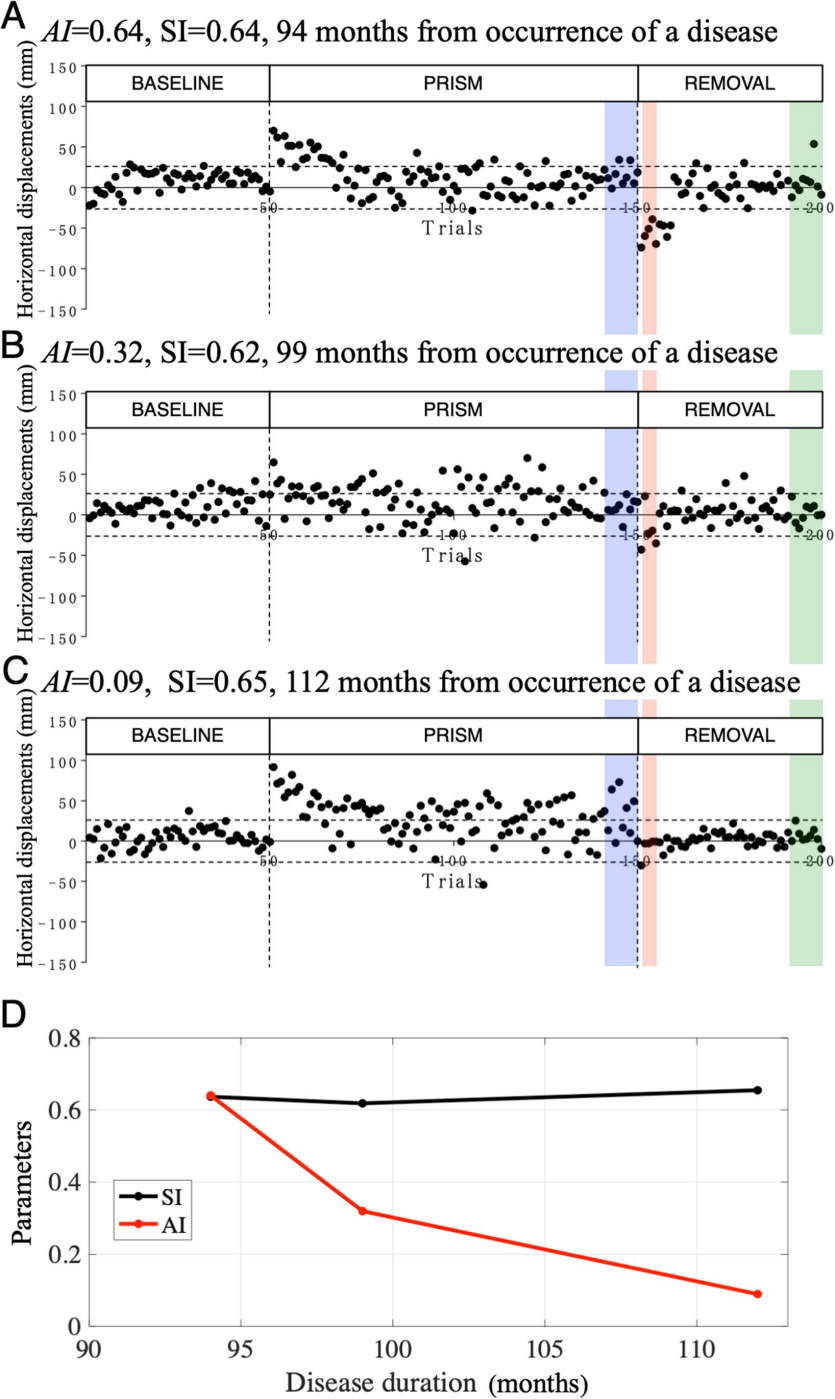
Example of prism adaptation of hand-reaching movement in a cerebellar patient (CD39 in Table 1). (**A**) First test data. (**B**) Second test data, obtained five months after the first test. (**C**) Third test data, obtained 13 months after the second test. (**D**) Time courses of *AI* and *SI* of this patient. The abscissa shows the follow-up duration (months) from the first test

The *AI* and SARA score of one SCA31 patient were 0.64 and 10 (*SI* = 0.64) (Table 1, CD39, the initial exam), respectively (Fig. 2A), suggesting that his motor learning capability was mildly impaired with mild ataxia. However, five months later, his *AI* and *SI* were 0.32 and 0.62 (Fig. 2B), and 13 months later, 0.09 and 0.65 (Fig. 2C), respectively. Thus, his *AI* decreased considerably rapidly, whereas his *SI* remained unchanged (Fig. 2D), suggesting that the impairment of motor learning started earlier than the progression of ataxia. We obtained similar results in five patients with MSA-P in previous studies [5].

### Changes in AI and SI with Disease Duration in each Disease Type of Cerebellar Degeneration

**Fig. 3:**
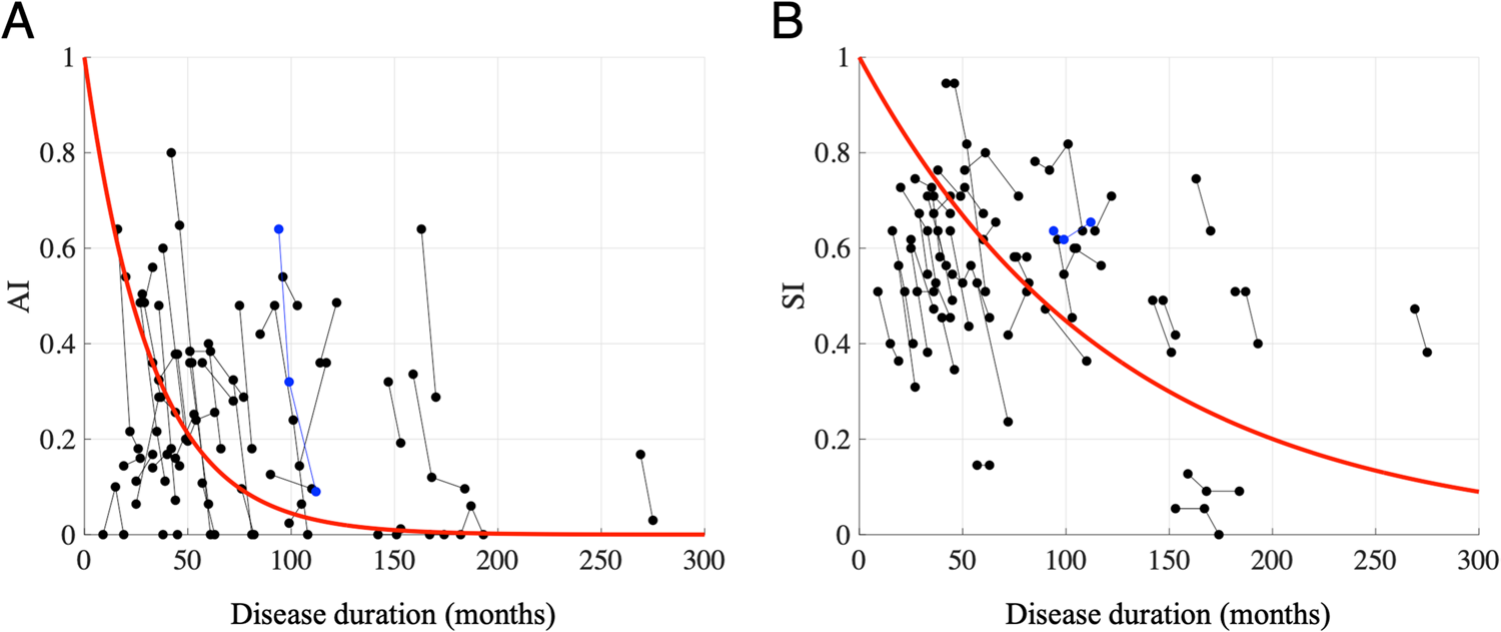
Changes in *AI* and *SI* with the duration of cerebellar degeneration. For (**A**) *AI* and (**B**) *SI* and the disease duration. Data were obtained from 40 cerebellar patients. The abscissa shows the disease duration. Dots connected with lines represent the data obtained from the same subject. Blue dots and lines show the data of the patient with CD39 in Table 1. Red curves show the best-fitted exponential curves overlaid on the raw data. The time constant of *AI* is 38.8 months and that of *SI* is 170.2 months

To see how *AI* and *SI* represent the temporal course of worsening of cerebellar disease, we explored the changes in *AI* and *SI* with the disease duration from the onset of symptoms in patients. Both *AI* and *SI* decreased over a long period of time as the disease duration increased (Fig. 3A and B). There was a significant negative correlation between *AI* and the disease duration (*R* =  − 0.33, *p* < 0.01) and between *SI* and the disease duration (*R* =  − 0.23, *p* < 0.05). Assuming from the pattern of the plots in Fig. 3 that both *AI* and *SI* exponentially decreased from one to zero as the disease duration increased, we fitted *AI* and *SI* with exponential curves (Eqs. 2 and 3) obtained by the least squares method (red curves in Figs. 3A and B). We calculated the time constants *a* and *b,* defined as the disease duration taken for *AI* and *SI* to decrease toward 0.368 [= exp (− 1)], respectively. They were 38.8 months (*a*) for *AI* and 170.2 months (*b*) for *SI*, indicating that *AI* decreased four times more rapidly than *SI*.

**Fig. 4:**
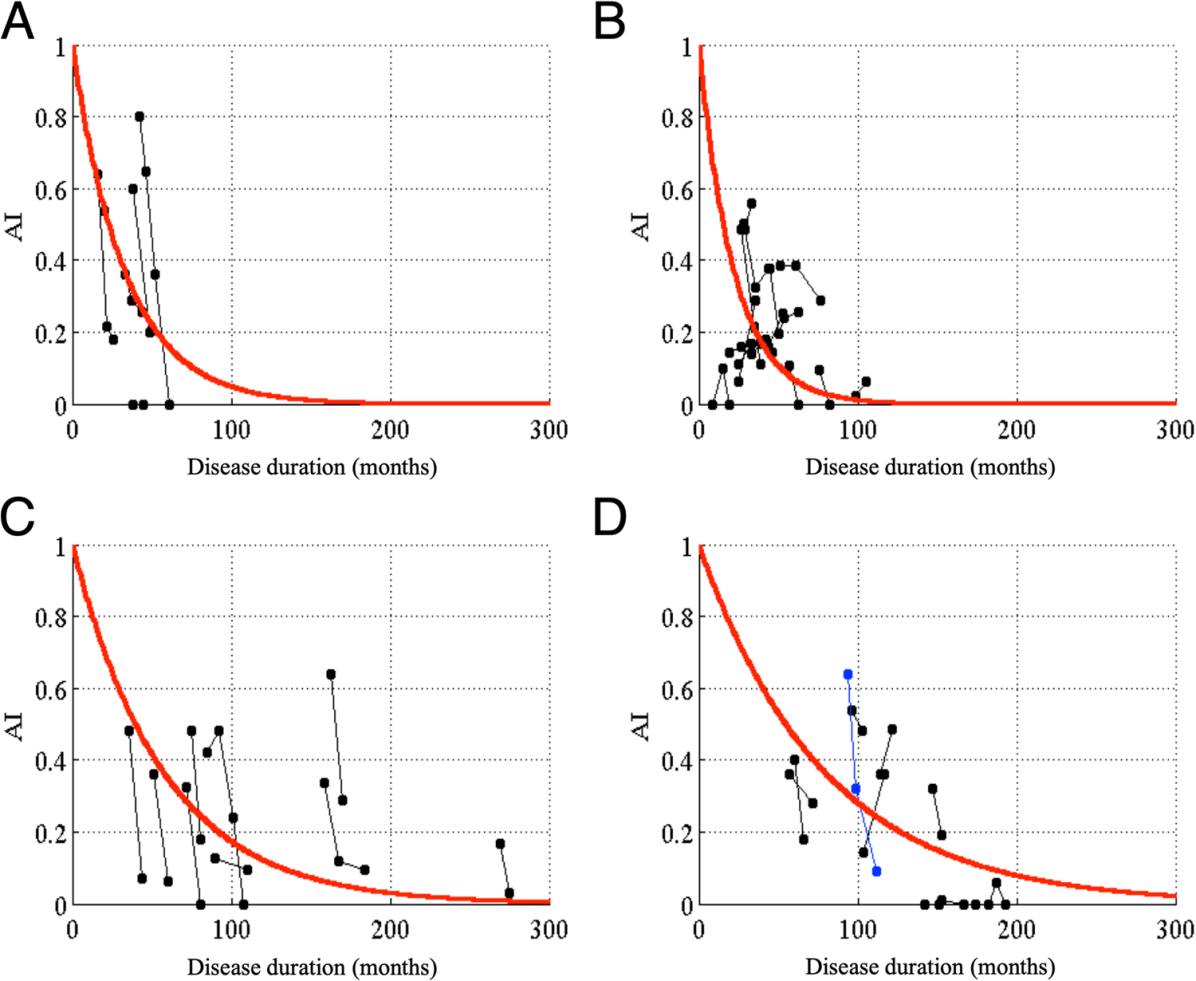
Changes in *AI* with the disease duration in different types of cerebellar degeneration. For (**A**) MSA-P, (**B**) MSA-C, (**C**) MJD, and (**D**) SCA6 or SCA31. Conventions are the same as those in Fig. 3. The time constants of *AI* (*a*) are 26.9, 39.8, 68.9, and 95.0 months in the patients with MSA-P, MSA-C, MJD, and SCA6 or SCA31, respectively

**Fig. 5:**
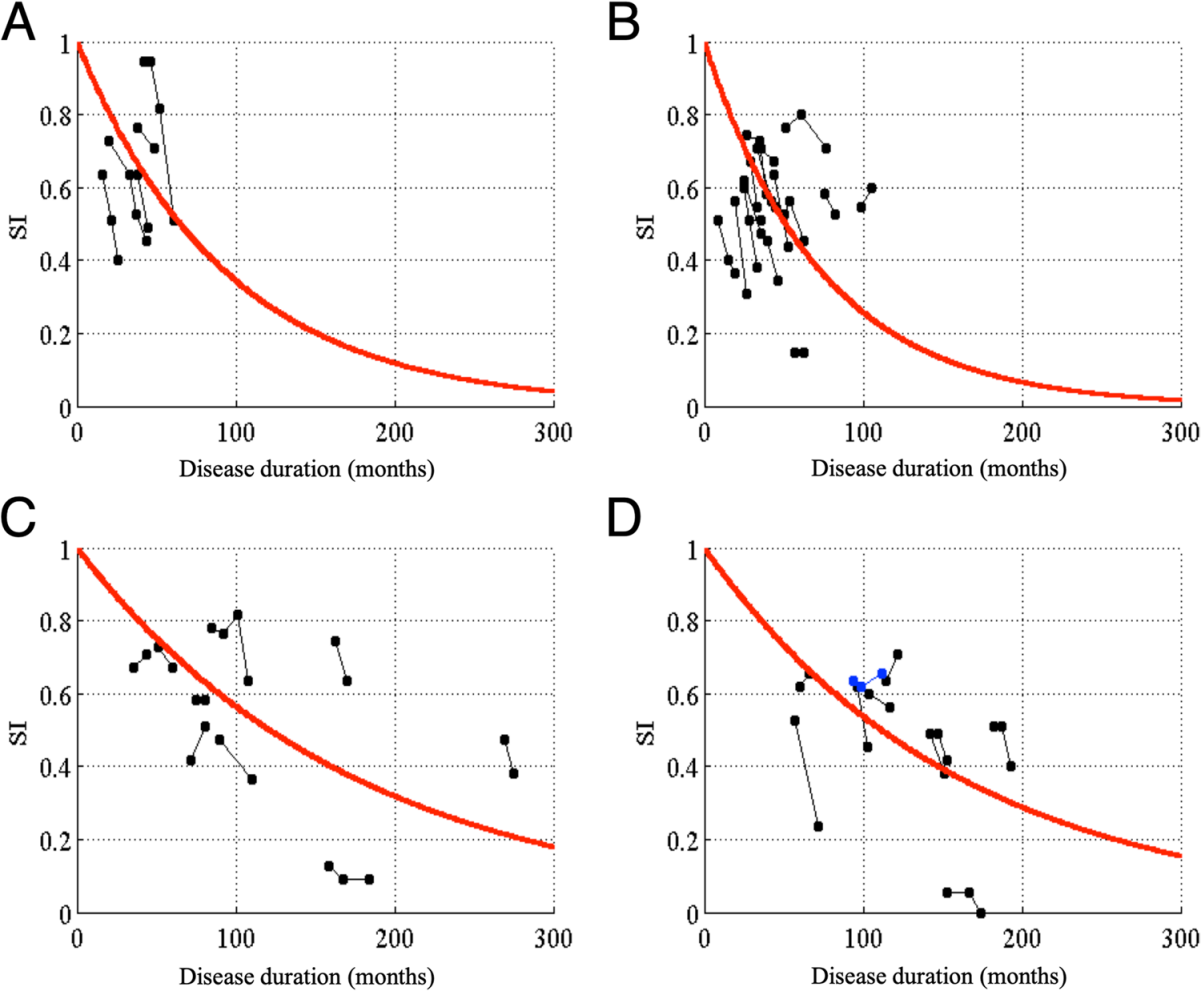
Changes in *SI* with the disease duration in different types of cerebellar degeneration. For (**A**) MSA-P, (**B**) MSA-C, (**C**) MJD, and (**D**) SCA6 or SCA31. Conventions are the same as those in Fig. 3. The time constants of *SI* (*b*) are 88.8, 112.9, 210.7, and 193.8 months in the patients with MSA-P, MSA-C, MJD, and SCA6 or SCA31, respectively

We also showed changes in *AI* (Fig. 4) and *SI* (Fig. 5) with the disease duration in each disease type. Using the above calculation, we found that the time constants of *AI* (*a*) were 6.9 months for MSA-P, 39.8 months for MSA-C, 68.9 months for MJD, and 95.0 months for SCA6 and SCA31. On the other hand, the time constants of *SI* (*b*) were 88.8 months for MSA-P, 112.9 months for MSA-C, 210.7 months for MJD, and 193.8 months for SCA6 and SCA31. These results indicate that both *AI* and *SI* of the non-hereditary cerebellar degenerative diseases (MSA-P and MSA-C) decreased more rapidly than those of hereditary cerebellar degenerative diseases (MJD, SCA6, and SCA31), consistent with their clinical course [10, 11]. In addition, *AI* decreased more rapidly than *SI* in any of the four types of cerebellar degeneration. To summarize, *AI* reflected disease deterioration more sensitively than *SI* in the early stage of any cerebellar degenerative disease.

### Relationship between AI and SI

**Fig. 6:**
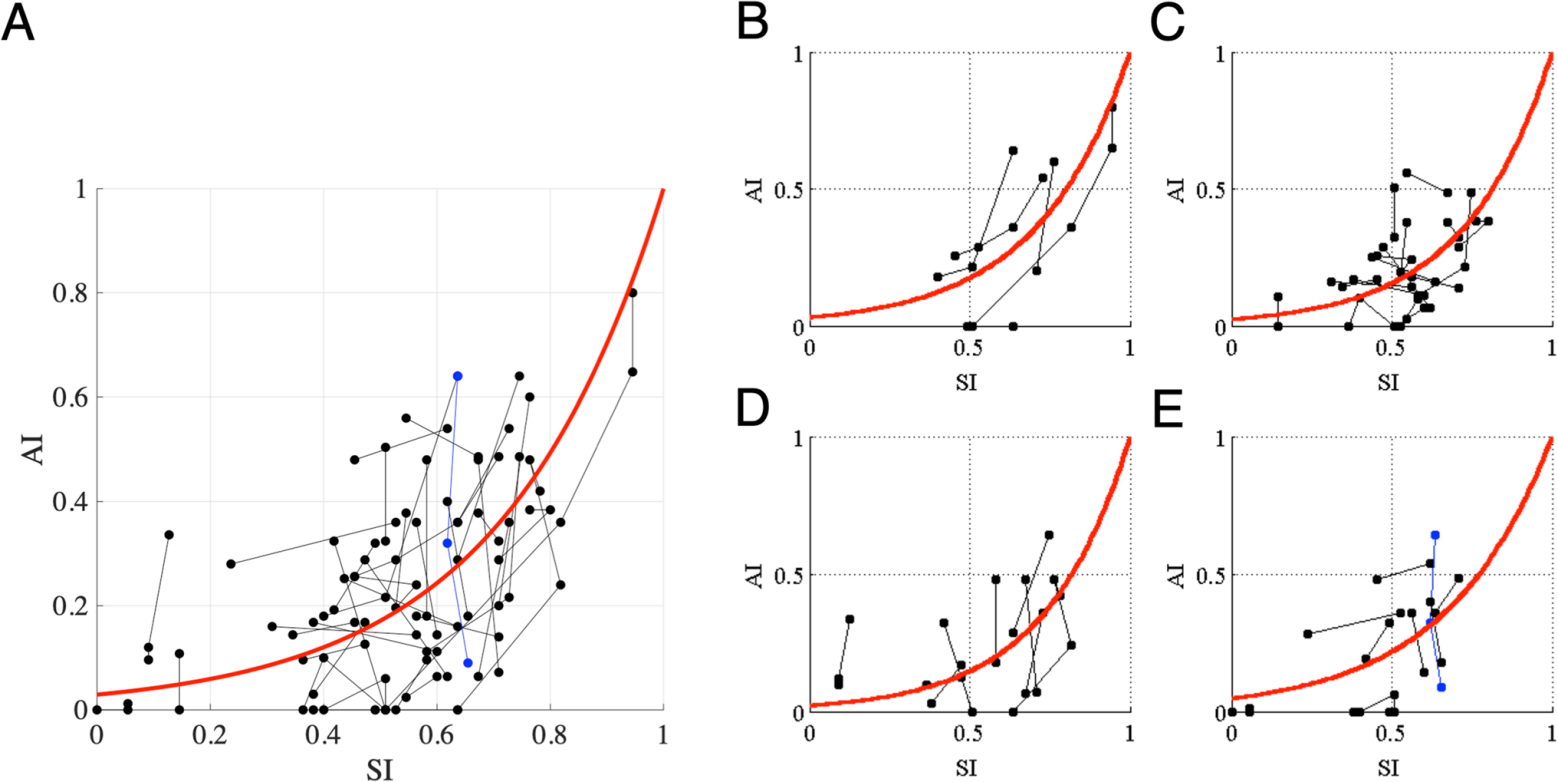
Relationship between *AI* and *SI*. For (**A**) all patients, (**B**) MSA-P, (**C**) MSA-C, (**D**) MJD, and (**E**) SCA6 or SCA31 patients. The abscissa shows *SI* and the ordinate shows *AI*. Dots connected with lines represent the data obtained from one patient. Blue dots and lines show the data for CD39 in Table 1. A red line shows a best-fitted exponential curve overlaid on the raw data. The amounts of decrease in *SI* [= (1-*SI*)] when *AI* decreases from 1 to 0.368 [= exp (-1)], the value of c in Eq. (4), are 0.326 in (A), 0.345 in (B), 0.323 in (C), 0.313 in (D), and 0.394 in (E)

Next, we focused on the relationship between *AI* and *SI* for all patients assuming *SARA* = 27.5 is the maximum score (Fig. 6A). Many *AI*s and *SI*s distributed within 0–0.64 and 0.35–0.82, respectively. None of the data points were observed in the upper left area of the graph with high *AI* and low *SI*, indicating that there was no such case with a high *AI* with a high SARA score. Namely, SARA score maintained low even if *AI* decreased. The value of *c*, defined as (1 − *SI*) when *AI* decreases from 1 to 0.368 [= exp (− 1)], was 0.326 in all patients (Fig. 6A), indicating that *AI* decreases from 1 to 0.368, whereas *SI* decreases from 1 to 0.674 (SARA score increase from 0 to 8.965). Essentially the same result was seen when admitting the highest value of 40 as the maximum SARA score instead of 27.5 (Supplementary Fig. 1).

We then examined the relationship between *AI* and *SI* in each disease type of cerebellar degeneration. Positive correlations between *AI* and *SI* (*R* = 0.47, *p* < 0.01) were consistently seen in all diseases (Figs. 6B-E). Compared to the value *c* of 0.326 when all the patients were calculated, they were 0.345 for MSA-P, 0.323 for MSA-C, 0.313 for MJD, and 0.394 for SCA6 and SCA31, with the mean and SD being 0.344 ± 0.036, indicating that the relationship between *AI* and *SI* was commonly seen in all the disease types examined in this study.

**Fig. 7:**
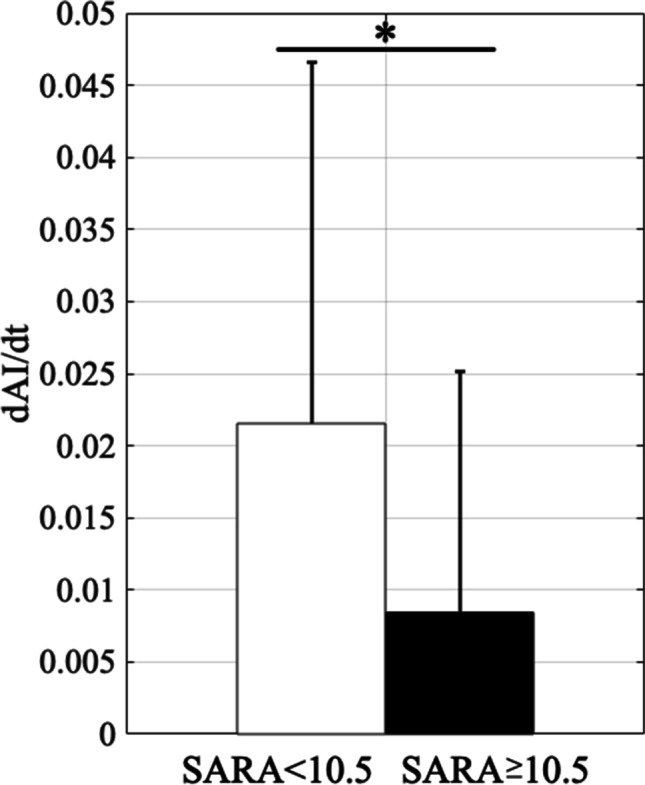
Change rates of *AI*. The averaged change rates of *AI* are 0.022/month when SARA scores are lower than 10.5 (*SI* < 0.6) and 0.008/month when SARA scores are higher than 10.5 (*SI* ≥ 0.6). * *p* < 0.05 by *t*-test. Error bars represent SD

Finally, we focused on the relationship between the change rate of *AI* (*dAI/dt*) and SARA score. Our 56 data on *dAI/dt* are divided into two groups of 28 data above and below the SARA score of 10.5. The disease state is severe when SARA scores were ≥ 10.5 (The *SI* at *SARA* = 10.5 is 0.6). The averaged *dAI/dt* was 0.022/month when SARA scores were < 10.5, and 0.008/month when SARA scores were ≥ 10.5 (Fig. 7). Thus, there was a significant difference in the change rates of *AI* between low (SARA scores < 10.5) and high (SARA scores ≥ 10.5) SARA scores (*p* < 0.05, two-sample t-test, SARA score = 10.5). Similar tendencies were observed at different thresholds of SARA scores around 10.5, although numbers of sample sets were skewed depending on the threshold (Supplementary Fig. 2). For example, significantly smaller *dAI*/*dt* were observed at SARA score = 10 and 11.5, though the numbers of samples were not even as in SARA score = 10.5 (SARA score = 10: 32 intervals vs 24 intervals; *SARA* = 11.5: 24 intervals vs 32 intervals). Overall, these findings suggest that patients’ motor learning capabilities were largely impaired before the patients show SARA scores ≥ 10.5.

## Discussion

In this study, *AI,* calculated from the data from prism adaptation in the hand-reaching task, decreased rapidly before SARA scores increased in 40 patients with cerebellar degeneration. While we have to admit that the numbers of patients and intervals we tested were limited, we could observe consistent *AI* decrease during the disease course in all four disease groups (MSA-P, MSA-C, MJD, SCA6 and SCA31). In addition, the decrease of *AI* and the increase of SARA score were more obvious in patients with non-hereditary cerebellar degenerative diseases (MSA-C and MSA-P) than in the hereditary cerebellar degeneration (MJD, SCA6, and SCA31). Thirdly, we observed that *AI* decreased rapidly whereas SARA score was not so high, and SARA score became high after *AI* became considerably low. Future studies by analyzing larger numbers of patients and prism-adaptation testing would allow us to obtain more accurate *AI* decrease curve in each disease.

### Quantitative Evaluation

Clinical scales such as ICARS [2] and SARA [3] have been developed and used in routine medical examinations. However, it is very difficult to diagnose, evaluate, and treat neurodegenerative diseases correctly by using only these two scales, because the SARA score is a subjective measure and depends on the experience and skill of the examiner. Furthermore, their changes are very subtle such as only approximately one point per year in SCA6 [12–15]. Likewise, these diseases progress very slowly in many SCA cases. A change of one point in SARA score can occur by placebo effect [16]. Therefore, a more quantitative and sensitive method is required in evaluating cerebellar function. In addition of SCA cases, this is a very important issue for rare neurodegenerative diseases of the cerebellum [1].

To enable us to assess disease progressions, development of a quantitative method has been attempted for evaluating ataxic gait, upper limb ataxia, or both in patients with cerebellar degenerative diseases by using novel technologies of accelerometers [17], a robot device including pen-like parts [18], and an infrared sensor [19], respectively. As the motor learning is one of the essential function of the cerebellum, we consider it important to include prism adaptation for a quantitative assessment of cerebellar dysfunction. In this regard, we previously developed a method using *AI* for evaluating cerebellar motor learning function by using touchscreen technology, and showed that 5 MSA patients gradually became incompetent for adapting to the task while their disease progressed [5]. The present study extended our observation on each patient including those with SCAs, and confirmed that the *AI* decreases with time in all cerebellar degenerations (Fig. 3A). We also found that the *AI* in patients with cerebellar degeneration tend to decrease more rapidly than their *SI* (Fig. 2D). Although this observation can be useful in clinical situation, it should be noted that *AI* and *SI* reflect different physiological brain functions. *SI* reflects ataxias of limbs and speech as well as those of balance, while *AI* is based on motor learning capability. In order to compare the decrement speeds of these two parameters, we need to precisely map each physiological function in the cerebellum, know which part of the cerebellum is affected in disease conditions, and point out when clinically manifests. Nevertheless, the present study suggested that motor learning may be a useful point for detecting the cerebellar dysfunctions. In fact, the *AI* decrease was more dramatic in earlier stage than in later stage (Fig. 7).

### Relationship between Motor Learning and Ataxia

A large number of animal studies using experimental paradigms of ocular reflex adaptations and eyeblink conditioning have suggested that the cerebellum plays a crucial role in motor learning [20, 21]. Long-term depression and potentiation at Purkinje cell synapses, originally proposed by Marr [22] and Albus [23], and demonstrated by Ito’s group [24–28], are assumed to underlie cerebellar motor learning [29].

Patients with cerebellar degenerative diseases showed impaired motor learning of voluntary forelimb movements [e.g., [4, 5, 7, 30, 31]]. In forelimb movements, cerebellar learning is assumed to be used to update the internal model of a movement acquired through cerebellar learning [8, 32, 33]. For example, we can autonomously touch our finger to the tip of our nose without any visual guide in finger-to-nose test. In our daily life, we repeatedly touch our finger to the tip of our nose from childhood. The distance of finger movement needed to touch the tip of the nose depends on the length of the arm and the height of the body. Repetition of such a finger touch induces cerebellar function to learn to adjust the internal model of the finger touch movement to the current body state [8]. The internal model thus formed in the cerebellum is assumed to enable us to move our forelimb to the target, i.e., the tip of our nose, in the feed-forward manner throughout our life [8, 34]. Since the impairment of motor learning represented by *AI* became evident much earlier than the progression of ataxia represented by SARA score, the impaired cerebellar motor learning of the internal model of movement may be the cause of the ataxia, but not the result of the ataxia.

### Rehabilitation for Patients with Cerebellar Degeneration

Several different views are addressed on the significance of physiotherapeutic training for the patients with cerebellar degeneration. For example, Ilg et al. [35, 36] reported that intensive and continuous coordinative training led to short- and long-term improvements of motor performance in patients with cerebellar degeneration. On the other hand, Aprigliano et al. [37] reported that although patients with cerebellar ataxia could stabilize their gaits by repetition of gait training on the treadmill moving at a constant velocity, they failed to stabilize their gaits against the repeated presentation of perturbations in treadmill velocity, indicating that the prediction of the occurrence of perturbations by the cerebellum was impaired in these patients.

Because the *AI* value of a patient with cerebellar degeneration was high when his/her SARA score was lower than 10.5 (Fig. 6), rehabilitation training by cerebellar learning to improve motor function (e.g., gait and forelimb movement, etc.) may be effective for this patient. However, when his/her SARA score was lower than 10.5, his/her *AI* value decreased largely with time (Fig. 7). Future research will be expected to clarify whether his/her *AI* can be increased or maintained by rehabilitation training. On the other hand, rehabilitation training by cerebellar learning might not be very effective for patients with SARA scores higher than 10.5 but with low *AI*s (Fig. 6). Thus, to design a rehabilitation strategy for patients with cerebellar degeneration, it is important to evaluate how much of the motor learning capability is available by referring to their *AI*.

## Conclusion

We evaluated motor learning and ataxia at intervals of several months in 40 patients with cerebellar degenerative conditions. We found that *AI* indicating cerebellar motor learning decreased most markedly in both MSA-C and MSA-P, moderately in MJD, and mildly in SCA6 and SCA31. Overall, *AI* decrease occurred more rapidly than the SARA score increase. *AI* is a useful marker for both rapid and indolent progressions in cerebellar diseases, and that evaluating the motor learning of patients can be particularly valuable for detecting cerebellar impairment, which is often masked by parkinsonisms and other signs.

### Supplementary Information

Below is the link to the electronic supplementary material.

Supplementary file1 (DOCX 252 KB)
