# Supplementary material for: Temporal Relationship between Impairment of Cerebellar Motor Learning and Deterioration of Ataxia in Patients with Cerebellar Degeneration
Source: Cerebellum. 2023 Apr 28;23(4):1280–92. doi: 10.1007/s12311-023-01545-1 (PMC11269492; doi:10.1007/s12311-023-01545-1)
Supplement: Supplementary file 1 — Supplementary file1 (DOCX 252 KB) [file 12311_2023_1545_MOESM1_ESM.docx]

**Supplementary Fig. 1** Relationship between AI and SI for all patients when SI was additionally calculated assuming that SARA can range from 0 to 40.


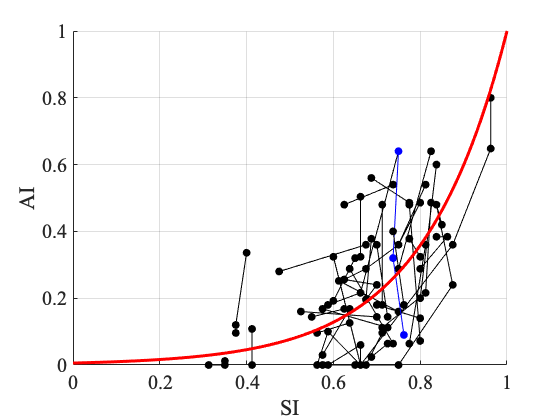


Conventions are the same as those in Fig. 6A.

.

**Supplementary Fig. 2** Change rates of *AI* with variable thresholds of SARA scores.

**
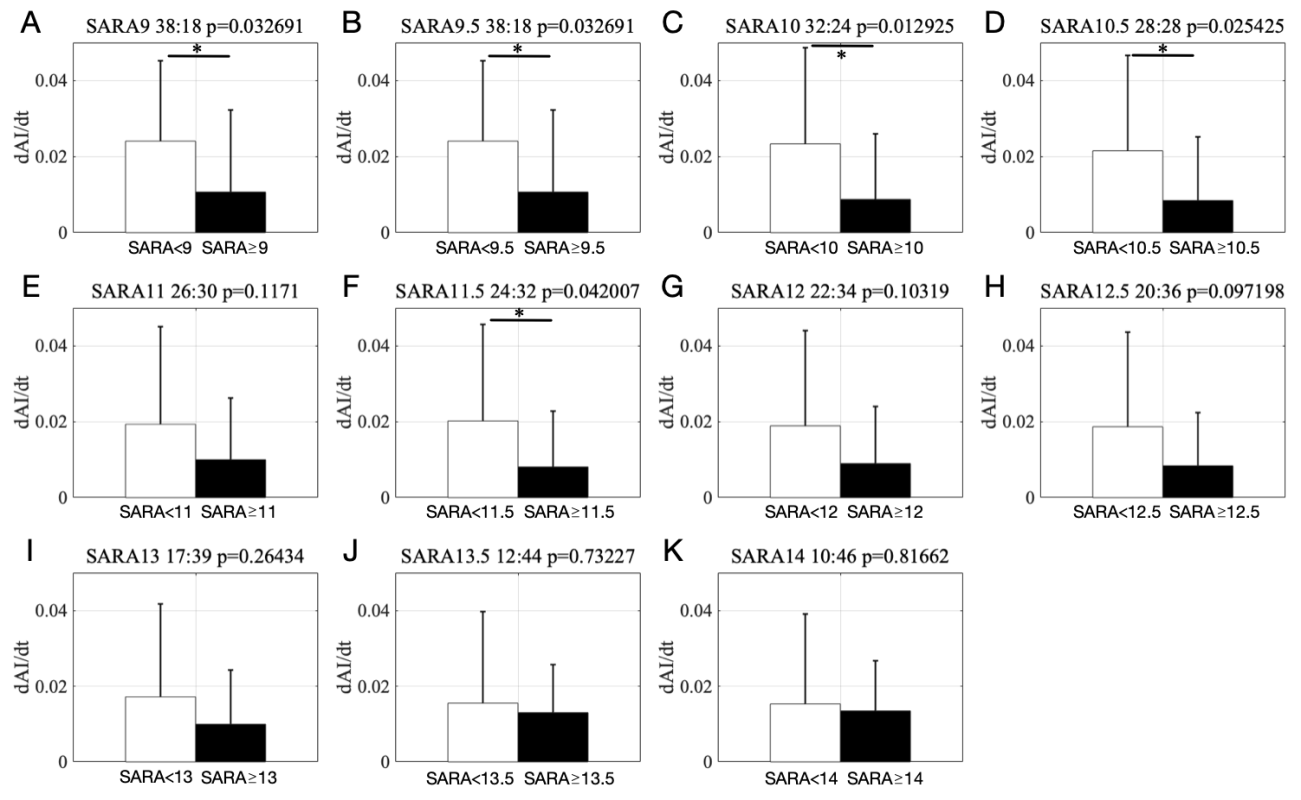
**

Change rates of *AI* were dependent on the SARA scores in SCD patients. Change rates of *AI* were compared among 56 patients at the stage when their SARA scores were between 9 and 14. The top of each graph shows the SARA score and the number of patients compared, and *p*-values are shown from left to right. * *p*< 0.05 by t-test. Error bars represent SD.
